# Acknowledgment to Reviewers of *Biology* in 2021

**DOI:** 10.3390/biology11020226

**Published:** 2022-01-30

**Authors:** Biology Editorial Office

**Affiliations:** MDPI AG, St. Alban-Anlage 66, 4052 Basel, Switzerland

Rigorous peer-reviews are the basis of high-quality academic publishing. Thanks to the great efforts of our reviewers, *Biology* was able to maintain its standards for the high quality of its published papers. Thanks to the contribution of our reviewers, in 2021, the median time to first decision was 16 days and the median time to publication was 37 days. The editors would like to extend their gratitude and recognition to the following reviewers for their precious time and dedication, regardless of whether the papers they reviewed were finally published:
Aalto, DanielLázaro-Diéguez, FranciscoAbakumov, EvgenyLazzari, RafaelAbbasi, Arshad MehmoodLe Ferrand, HortenseAbdeen, AhmedLe Goff, Gaëlle Abdel-Daim, Mohamed M.Le Joncour, VadimAbdel-Latif, Hany M. R.Le Questel, Jean-yves Abdelmagid, SalmaLecaille, FabienAbdović, SlavenLederer, Ann-KathrinAbé, HiroshiLederer, EleanorAbedelmalek, SalmaLee, Chung-TaAboal, MarinaLee, Eun KyungAbondio, PaoloLee, Eun-WooAbrantes, Ana MargaridaLee, Heng-JuAcevedo, Juan JoseLee, IsaacAcosta-Avalos, DanielLee, JaeminAdamakis, Ioannis-DimosthenisLee, JiyoungAdamek-Urbańska, DobrochnaLee, KangwonAdamowicz, ElizabethLee, KwanukAdams, Brian D.Lee, NatuschkaAdamska, AleksandraLee, SangmiAddamo, Anna M.Lee, Seung HwanAddy, Partha SarathiLee, SeungyeoAdnan, MdLee, Tse-MinAdorni, FulvioLee, Tsung-HsienAdur, MalavikaLee, Tzong-ShyuanAdvani, VivekLee, Yuan-TiAfanou, Anani K.Lee, Yu-ChiAfrashtehfar, Kelvin IanLegalov, AndreiAgbaje, OluwatoosinLegaz, IsabelAghajanyan, AnushLeherte, LaurenceAguado, JulioLeibowitz, AvshalomAhlstrøm, ØysteinLeonardi, AdrijanaAhmadi, SohaLeone, Vanessa A.Ahmed, IftekharLerma, ClaudiaAhmed, MukhtarLeslie, JackAhmed, TofayelLeso, LorenzoAidar, Felipe J.Lessios, Harilaos A.Ajigboye, Olubukola O.Lessl, JasonAkada, JunkoLeu, Jiann-HorngAkakpo, RolandLev, SophieAkbulut, SamiLevitskii, SergeyAkkouch, AdilLexa, MatejAlaimo, AlessandroLi, ChenshuangAlam, MobashwerLi, DongshengAlber, AndreasLi, GuohuiAldred, KatieLi, HanAldrich, Melissa B.Li, JixuAlevi, Kaio Cesar ChaboliLi, Shengwen CalvinAlex, AnoopLi, ShuqiAlexander, Francis Borgio J.Li, TengAlfonso, SébastienLi, YuxinAli, Lamiaa M. A.Liao, Chun-HouAli, NawabLiao, Pin-ChaoAlisaac, EliasLiesebach, HeikeAlizargar, JavadLightfoot, Adam P.Al-Kharaz, Ali A.Ligtenberg, A.J.M.Allen, EdithLiguoro, DominiqueAllen, TanuLim, Tae-GyuAllison, Simon J.Lima Do Monte-Neto, Rubens Al-Maharik, NawafL’Imperio, VincenzoAlmeida, RaquelLin, Guo-HaoAlmouzni, GenevieveLin, LiAl-Mubarak, Bashayer R.Lin, Myat T.Al-Mutairi, NawafLin, Nai-ChunAlonso, JavierLin, Po-HanAlptekin, BurcuLin, TaoAlqarni, Abdulaziz SaadLio, DomenicoAl-Rimawi, FuadLiobikas, JuliusAlsaab, HashemLionetto, Maria GiuliaAl-Shargabi, Amal A.Liordos, VasiliosAltieri, SaverioLipari, DarioAltruda, FiorellaLiska, FrantisekAlvarez, Olga GarciaLista, Florigio RomanoÁlvarez-González, Carlos AlfonsoLiu, Cheuk LunÁlvarez-Mercado, Ana I.Liu, Chung-JungAlves, C. HenriqueLiu, EnqiAlves, Ricardo CastroLiu, Eric MinweiAlves, ThaisLiu, FangyuAlzubaidi, LaithLiu, HongweiAmadio, PatriziaLizandra, JorgeAmalfitano, StefanoLješević, MarijaAmbrožič-Dolinšek, JanaLjiljana, KrstinAmero, CarlosLloret, María Pilar PecciAmiridis, GeorgeLo Furno, DeboraAmirkhani, MasoumeLoiola, Rodrigo A.Ammar, AchrafLok, Hiu ChuenAmoah, IsaacLombardi, Vincent C.Amoutzias, GrigorisLong, Cheng-YuAn, ChunjiangLonghitano, YaroslavaAnagnostopoulos, IoannisLoo, ShiningAnand, UttpalLoosli, FelixAnatolevich, Chistyakov VladimirLopes, IsabelAnderson, KevinLópez, Elena SánchezAnderson, Kirk E.López, Jesús AdriánAnderson, O. R.López, José I.Andersson, Maria A.Lopez, Jose JavierAndrade, ArturoLópez-Alvarez, DianaAndrade, José PedroLopez-Dolado, ElisaAndréani, JulienLópez-Gil, José FranciscoAndreucci, ElenaLoppi, StefanoAndrews, Melissa R.Lorenc-Koci, ElżbietaAnnona, Giovanni (Ivan)Lorkiewicz, Pawel K.Ansari, Mohd NazamLouca, VasilisAntal, LászlóLoukopoulos, PanayiotisAntonakopoulos, NikolaosLoureiro, Joana R.Antonelli, AlessandroLövei, Gabor L.Antonopoulou, EfthimiaLoveland, Jasmine L.Anver, ShajahanLoveridge, JoelAppel, JensLow, Wai YeeApu, Ehsanul HoqueLozniewski, AlainAragon, MarcelaLozo, JelenaArata, ToshiakiLu, HuiAraujo, RicardoLu, Ming-WeiArellanes-Robledo, JaimeLu, YouArendt, Lisa M.Luci, IsabelArenz, BrettLuciani, ValeriaArias, AndresLucini, DanielaArizmendi, Constancio MiguelLucke-Wold, BrandonArmento, AngelaLuis, AnaArokiyaraj, SelvarajLumsden, John S.Arora, Gurpreet KaurLuna-Perejón, FranciscoArrebola, Francisco A.Lund, IvarArrigoni, ChiaraLund, SandraArroba, Ana I.Luo, Kai-JunArruga, FrancescaLupashin, VladimirArslan, MuhammadLupi, Saturnino MarcoArsoniadis, Gavriil GeorgeLusa, MerjaArtunc, FerruhLuschi, PaoloArtym, JolantaLuther, Anne-MarieArufe, María C.Lutnicki, KrzysztofAsakawa, MitsuhikoLuttermann, ChristineAsdaq, Syed M.B.Luxton, G. W. GantAshley, JonLymperopoulos, AristotelisAshok, AjayLynch, Jeffrey JamesAshour, Mohamed LotfyMa, FengyunAshworth, Matt P.Ma, KesenAsnaghi, ValentinaMa, San-YuanAsnicar, DavideMaçãs, BenvindoAssinck, PeggyMachida, ShuichiAste, GiovanniMacias, AlvaroAstiz, SusanaMaciejczyk, MarcinAstl, JaromirMaciver, SutherlandAthanasiou, Labrini V.Mackay, Anson W.Atique, UsmanMadduri, SrinivasAttia, Ahmed SherifMademli, LidaAttri, KuldeepMademtzoglou, DespoinaAtukorallaya, DeviMaeda, NoriakiAtzori, LauraMagalhães, João P.Aubourg, SantiagoMägi, MarkoAudia, Jonathon P.Magiorkinis, EmmanouilAudisio, Marcela CarinaMahadik, BhushanAuger, PierreMahajan, GautamAuvynet, ConstanceMahata, SushilAvendaño, Dionisio F. Yáñez Mahmoud, Lobna BenAyala, JuanMajewska, MałgorzataAyayee, Paul AkwetteyMakaroff, ChristopherAyon, RamonMakrakis, SergioAzam, Aa HaerumanMalik, AdeelAziz, Abdul RashidMalinzi, JosephAzizi, HosseinMallela, Shamroop KumarBabenko, Vladimir NikolaevichMallo, NataliaBabiuk, ShawnMalmström, JohanBabkin, Igor V.Maloberti, AlessandroBaccarani, MicheleMalonia, SunilBachevalier, JocelyneMamen, AsgeirBachmann, FriederikeMamidi, MuraliBąchor, RemigiuszMamlouk, Amir MadanyBacova, Barbara SzeiffovaMamouni, KenzaBadiani, MaurizioMancarella, CaterinaBadr, AbdelfattahMancuso, MoniqueBae, Eui WonManenti, AntonioBagi, FerencMangeot, PhilippeBagyinszky, EvaMangiagalli, MarcoBahmad, HishamMangir, NaşideBajgain, PrabinManhart, MichaelBajoghli, BaubakManicone, Paolo FrancescoBal, WojciechManier, Sascha K.Balbach, MelanieManino, AuloBalcerczyk, AnetaMannell, HannaBaldanzi, GianlucaManni, GianlucaBaldi, ElisabettaManni, GiorgiaBaleba, Steve B. S.Manor, UriBalestra, ConstantinoManoylov, Kalina M.Balieva, Flora N.Manrique, EstebanBaloni, PriyankaMansor, Roman FouadBaltag, EmanuelMansueto, GelsominaBaltoumas, FotisMantzoukas, SpiridonBanach, ArturManuel, MarinBandiera, AlessandroManzanilla, RosaBandyopadhyay, SushobhanaManzo-Merino, JoaquinBara, JeffreyMaranesi, MargheritaBarabino, EmanueleMarchetti, PhilippeBaranowska-Wasilewska, MarlenaMarconi, Guya DilettaBarbalho, Sandra MariaMaréchal, AlexandreBarbaro, AnnaMarinelli, LucaBarbato, RobertoMarini, Herbert RyanBarberá, Gonzalo G.Marino, RosannaBarclay, SarahMarjanović, HrvojeBarinova, SophiaMarkmann, MariusBarnes, WilliamMarković, OliveraBarone, RobertoMarquardt, TomaszBarr, Stephen D.Marques, Ana CoelhoBarrero, María JoséMarques, IsabelBarros-García, DavidMarquetand, JustusBârsan, NarcisMarquez-Rocha, Facundo J.Bartish, Igor V.Marrasé, CèliaBartol, Frank F.Marrs, James A.Bärwolff, GünterMartel, CatherineBasak, SujitMartell, MariaBaschieri, FrancescoMartelli, GiuseppeBasco, LeonardoMartiel, Jean-LouisBashan, Luz E.Martin, FranciscoBasile, GiorgioMartin, KarinBasoli, ValentinaMartín, María P.Bassi, RobertoMartinez, DiegoBateman, AndrewMartínez, Víctor A.Bateman, Kelly S.Martínez-Álvarez, ÓscarBates, JohnMartinez-Amezcua, PabloBattail, ChristopheMartínez-Banaclocha, Marcos ArturoBaumann, AnjaMartínez-Esteso, María JoséBeamonte, Roberto MartínezMartínez-Guardado, IsmaelBeauchaud, MarilynMartínez-Navarrete, GemaBebbere, DanielaMartinez-Pastor, FelipeBeck, AndreasMartínez-Rodrigo, AbelBeck, Michael W.Martínez-Rodríguez, GonzaloBecker, DoreenMartínez-Soler, FinaBedada, GirmaMartins Júnior, Paulo AntônioBedini, GianniMartins, Airton C.Beedessee, GirishMartins, AndrewBeer, MeinradMartins, Jorge N. R.Begemann, GerritMartins, MariaBegović, LidijaMartsenyuk, VasylBehnke-Borowczyk, JolantaMartz, FrancoiseBehr, MarcMaruyama, HaruhikoBehringer, ErikMarx, AlexanderBeine-Golovchuk, OlgaMas, FloreBekeschus, SanderMasaki, TakayukiBela, KrisztinaMascia, GiuseppeBellé, CristianoMascitti, MarcoBelluzzi, ElisaMasiulis, MariusBeloukas, ApostolosMasthoff, MaxBender, KellyMastinu, PierfrancescoBenissa, SaidMastore, MaristellaBenito, PedroMasucci, FeliciaBennardo, FrancescoMasucci, GiuseppeBennardo, LuigiMatafome, Paulo N.Bennett, HunterMata-Lima, HerlanderBenowitz, Kyle M.Matei, IoanaBentley, Robert F.Mateos, Pilar FernándezBeňuš, RadoslavMateussi, Nadayca T. B.Berchialla, PaolaMathiyalagan, PrabhuBercsényi, MiklósMatilla, Angel J.Berezin, AlexanderMatny, OadiBerg, Rune W.Matos, José Bernardo NoronhaBergamini, CarloMatsubara, KeiichiBergh, ØivindMatsubara, ShigekiBermejo, Ana MariaMattei, CésarBermejo-Poza, RubénMatúšková, MiroslavaBernabéu, CarmeloMatysiak-Kucharek, MagdalenaBernard, Ernest C.Matzner, Steven LeeBernardino, Raquel L.Mauceri, RodolfoBerríos, CristhianMaugeri, Andrea GiuseppeBertoglio, BarbaraMaurizio, AuroraBertolini, CamillaMawson, Peter R.Besmond, ClaudeMaximov, AlexeyBeyi, Ashenafi F.Maxwell, AnthonyBezu, LucilliaMayberry, ColleenBhagirath, AnjaliMazan-Mamczarz, KrystynaBhatia, HimanshiMcCarthy, Lynda H.Bhatia, Shashi KantMcElroy, KyleBhatia-Dey, NainaMcFadden, ClareBhattacharya, SanjoyMcKay, TinaBhawal, Ujjal KumarMcKeown, Peter C.Bi, LanrongMcLagan, DavidBiagi, MarcoMcLean, Kyle J.Biamonte, FlaviaMcMillin, MatthewBianchi, SerenaMcMurray, MichaelBianconi, FortunatoMeans, Robert T.Biasetti, PierfrancescoMeccariello, RosariaBiasin, ValentinaMechchate, HamzaBickford, NateMediano, Pedro A. M.Bicudo, AlvaroMeester, JosephinaBiernasiuk, AnnaMeghil, MohamedBilger, AndreaMehariya, SanjeetBilia, Anna RitaMehlhorn, HeinzBilic, IvanaMehrabian, Ahmad RezaBillerbeck, SaraMei, QichangBindels, LaureMeienberg, JanineBindu, Dhanesh SivadasanMeier-Gibbons, FrancesBirhanu, Biruk TesfayeMelanson, AlexandreBiró, BorbalaMénard, HervéBlamires, SeanMenck, Carlos FredericoBlanchet, GuillaumeMendes, Luis AndreBlank, SimonMendes, RafaelBlasco, LucíaMendez-Bermudez, AaronBlasco, Pilar SantolariaMeneguzzo, PaoloBlázquez-Castro, AlfonsoMeneses, CarlosBlomme, JonasMeng, WeihuaBobu, Livia IonelaMengarelli, AlessandroBoccalatte, FrancescoMenna-Barreto, Rubem Figueiredo SadokBocianowski, JanMenzo, StefanoBock, Niko C.Merah, OthmaneBocu, RazvanMercer, Derry K.Boeldt, Derek S.Mereiter, StefanBogdányi, Franciska TóthnéMeriggi, AlbertoBoiani, MicheleMerritt, DavidBokde, NeerajMesquita-Guimarães, JoanaBolcato, MatteoMessina, GiovanniBonato, MatteoMeštrović, TomislavBonato, MaudMetere, AlessioBonciu, ElenaMethenitis, SpyridonBondžić, Aleksandra M.Metovic, JasnaBonga, Sjoerd Evert WendelaarMettke-Hofmann, ClaudiaBoni, RaffaeleMetzler, DirkBonifacio, Maria AddolorataMeucci, Maria ChiaraBonsignore, Carmelo PeterMeulenbroek, Ruud G. J.Borczyk, BartoszMeunier, JoëlBordin, LucianaMhatre, SiddhitaBorg, BertilMichailidis, IoannisBorges, MargaridaMichałek, SławomirBorghesi, AndreaMichalet, RichardBorghini, AndreaMichalopoulos, Nikolaos V.Borkowska, Edyta MartaMichalski, MirosławBorland, SamanthaMickol, Rebecca L.Bornbusch, Sally LyonsMieog, Jos CorneliusBortz, EricMihalca, Andrei DanielBorzabadi-Farahani, AliMikolajczyk, AgataBoscari, ElisaMilardi, DaniloBoscutti, FrancescoMing, Li-juneBoshra, Hani Y.Minguez, PabloBosi, GiovannaMiniero, Daniela ValeriaBostan, HamedMinoura, AkiraBóta, AndrásMiotto, BenoitBotha, DeonaMirabella, GiovanniBoti, Michaela A.Miranda, AnaBouchet, AudreyMiranda-Sanchez, FabiolaBouet, ValentineMirón-Canelo, José-AntonioBoule, Jean BaptisteMirza, AhmadBoulé, Jean-BaptisteMishra, Amit KumarBou-Nader, CharlesMishra, Jay S.Bouskela, ElieteMishra, PriyankaBoussios, StergiosMisic, DusanBowman, Larry L.Miśta, DorotaBoyce, RichardMiszczyk, JustynaBoyd, Jonathan W.Mita, KojiBozic, JoskoMitchell III, Robert D.Bracco, EnricoMitchell, MichelaBracken, CameronMitić-Ćulafić, DraganaBraconi, DanielaMitsea, AnastasiaBranco, VascoMittapelly, PriyankaBrand, KorbinianMiura, HirokoBrandmayr, PietroMiyanishi, HiroshiBraquart-Varnier, ChristineMizrachi, DarioBrauer, Elizabeth K.Młynek, KrzysztofBravo, Susana B.Mo, Lein-RayBreedy, OdaliscaMoberg, LouiseBregadze, VladimirMocquet, VincentBrenciani, AndreaModos, DezsöBrestic, MarianMoe, Luke A.Brewer, GaryMogavero, Maria PaolaBrewster, Robert C.Mogk, AxelBrito, João PauloMognetti, BarbaraBriz-Redón, ÁlvaroMolenbroek, JohanBroccanello, ChiaraMolina, Cristina E.Brockmeyer, PhillippMolina-Espeja, PatriciaBrokaw, NicholasMoll, IngridBrown, Richard J. P.Momeny, MajidBrumatti, GabrielaMonari, AntonioBrundu, GiuseppeMondal, SanandaBrunelli, ElviraMondola, PaoloBruninga-Socolar, BethanneMonroe, Tanner O.Brunke, AdamMontag, DirkBruno, AlessandroMontaruli, GrazianoBruns, HeikoMontecucco, FabrizioBrut, MarieMontefusco, FrancescoBucher, EtienneMontemurro, NicolaBudde, HenningMora, FrancescoBūdienė, JurgaMoraes, Maria NatháliaBudzianowski, JanMorales, Benjamin OrtegaBudzier, HelmutMora-Medina, PatriciaBudzynska, SylwiaMorbidelli, LuciaBueno, Regiane Cristina Oliveira De FreitasMorcillo, RafaelBuitrago-Molina, Laura ElisaMoreira, HelenaBulut, ÖzgürMoreno-Perez, VictorBunc, VaclavMoretti, SamueleBunce, James A.Moretto, LuisaBurke, Susan J.Morgado, Mariza G.Burketova, LenkaMorgan, PaulBurlacu, AlexandruMorgenstern, DavidBurmester, AnkeMori, EmilianoBurnett, StephenMoriarty, TerenceBursić, VojislavaMoric, IvanaBurt, ChrisMorikawa, TakamichiBusca, AlessandroMorikawa, YukoBusch, AlbertMorimatsu, HiroshiBusoms, SilviaMoriondo, Andrea Butchbach, Matthew E. R.Moro, TatianaButera, AndreaMorozova, Vera V.Byeon, HaewonMorrison, Lloyd W.Byrnes, Catherine A.Mortara, LorenzoCacciola, FrancescoMorton, DavidCaceres, C. JoaquinMoschovi, MariaCáceres, IsabelMoșneguțu, Emilian FlorinCacialli, PietroMosqueira, MatiasCaeiro, Maria FilomenaMoss, Anthony G.Caesarendra, WahyuMöstl, ErichCafolla, DanieleMota, Manuel Galvão De Melo E.Cai, JiyangMotakis, EfthymiosCaimano, MiriamMotta, AntonellaCairo, StefanoMou, Hai-JinCal, MagdalenaMouli, RamasamyCaldara, RobertoMoura, TeresaCaldwell, JacobM.R., SanjayCalina, DanielaMugetti, DavideCalvo-Lobo, CésarMuharfiza, MuharfizaCâmara, Diogo RibeiroMuhovski, YordanCamilloni, GiorgioMukherji, AtishCammarisano, LauraMulens-Arias, VladimirCammilleri, GaetanoMulet, Jose M.Campa, Carlo CosimoMulherkar, ShalakaCampera, MarcoMüller, Marius N.Campioni, IlariaMüller, MartinCampos, Jonatan GarcíaMulligan, Vikram K.Candela, HéctorMuñiz-Ramirez, AlethiaCandiani, SimonaMuñoz, Rosa María López-PintorCangemi, GiulianaMuñoz-López, MónicaCangkrama, MichaelMünster-Wandowski, AgnieszkaCantamessa, SimoneMunuera, PedroCanu, LetiziaMurai, KojiCao, DongdongMurakami, TeruoCaparros-Martin, JoséMuravyov, Alexei V.Capela, João PauloMurphy, RonanCapillo, GioeleMurtas, GiuliaCapocasale, GiorgiaMuruganandan, ShanmugamCapoccia, MassimoMusacchia, FrancescoCapoccioni, FabrizioMusarella, Carmelo MariaCappa, FedericoMusgrave, Christopher Stephen AndrewCappai, Maria GraziaMusolin, Dmitry L.Cappelli, GianniMussi, ChiaraCaputi, LuigiMusumeci, GiuseppeCaratozzolo, MarianoMusumeci, RosarioCardinali, IreneMyakala, KomuraiahCardona, FernandoMylona, PhotiniCardone, Maria FrancescaMylonas, IoannisCardoso, Danon ClemesMymryk, JoeCardoso, Francisca AlvesMystkowska, JoannaCardoso, LuísNabi, NesrineCardoso, PauloNadappuram, Binoy PauloseCardoso, SusanaNadein, KonstantinCareau, VincentNagajyothi, Jyothi F.Carl, DanielNagasawa, KazueCarlberg, CarstenNagata, KojiCarlomagno, ChiaraNaidu, RakeshCarlucci, RobertoNaiki, HironobuCarmen, MejíaNainiene, RasaCarrer, AlessandroNakazawa, MitsuruCarril, JulietaNalbone, DavidCarrilho, EuniceNam, Ki HyunCarroll, PatrickNanda, SatyabrataCaruana, AmandineNardin, MatteoCarullo, GabrieleNarouei-Khandan, Hossein A.Caruso, GiuliaNashine, SonaliCarvalho, CristinaNäslund, JoacimCarvalho, MárciaNassar, NicolasCarvalho, Marta S.Nastuta, Andrei VasileCasadei, NicolasNat, Irina RoxanaCasas, AlejandroNatalini, AlessandroCasas-Barragán, AntonioNatarajan, SivaramanCascella, MarcoNatu, RuchaCaschera, FilippoNavarro, EstanisCassill, Deby L.Navarro, NicolasCastagnetti, StefaniaNavarro-Alvarez, NaluCastañeda, ArkaitzNavarro-León, EloyCastañeda, CarmenNavas-López, Víctor ManuelCastiglione, RobertoNavazio, AlessandroCastillo, AntonioNayak, DigantCastillo, PabloNees, MatthiasCastro-Munozledo, FedericoNejman-Faleńczyk, BożenaCastro-Vázquez, AlfredoNellimarla, SrinivasCatala, Maria MorenoNemeskéri, EszterCatalano, CaterinaNemeth, EllaCatanese, AlbertoNeri, TommasoCatanese, GaetanoNesher, NirCatar, RusanNethi, Susheel KumarCatoni, MarcoNeumann, JakeCauli, OmarNeuwirt, HannesCautela, DomenicoNeves, JoãoCava, ClaudiaNevola, RiccardoCavalca, LuciaNewman, Robert H.Caviglia, Gian PaoloNewton, Axel H.Cazzola, RobertaNg, Ashley P.Ceballos-Laita, LuisNgoy, KikomboCelestino, LeandroNguyen, Joe TruongCelie, PatrickNguyen, Quang D.Celsi, FulvioNicolussi, Paola S.Cerda, ArtemiNiculae, DanaCerenius, LageNielsen, Vance GirardCerritelli, GiuliaNii, TerukiCervera, AnaNijman, VincentCevasco, RobertaNikoh, NaruoChaber, RadosławNikolic, JovankaChahwan, RichardNikoloudakis, NikolaosChaki, MouniraNiku, MikaelChakrabarty, SwapanNimbs, Matt J.Chakraborty, SagnikNirala, Niraj K.Chakraborty, SumitNirengi, ShinsukeChakravarthi, V. PraveenNishijima, KazutoshiChallet, EtienneNishimura, NorihisaChamakura, Karthik R.Nishino, TomofumiChan, Elsa C.Nishio, MizuhoChandrasekaran, MurugesanNistor, EleonoraChang, Audrey N.Niyibizi, ChristopherChang, ChengNöbel, SabineChang, Chia-JungNoble, JohanChang, Ching-JinNoda, MamiChang, Hao-XunNogueira, TeresaChang, Ke-VinNombela, IvanChang, KoyinNoorani, ImranChang, Nai-JenNorimatsu, YutaChang, Wen-WeiNormand, AliceChao, Yun-YangNorton, Amie E.Chao, ZhiNotter, MarkusChapman, NadineNourbakhsh, MahtabChardon, FabienNovikov, AlexanderCharles, Christopher J.Nowak, AnnaCharmley, EdNowicka, GrażynaCharneca, Rui Miguel CarrachaNowinska, AnnaCharoenngam, NipithNoyes, Richard D.Chartosia, NikiNtzimani, AthinaChatla, KamalakarNunes, MonicaChatterjee, IshitaNunziata, AngelinaChatterjee, PayelNuti, MarcoChatterjee, PoulamiNyabuga, FranklinChatterjee, SurajitObermayer, AstridChaturvedi, DeeptiOcchipinti, AnnaChavanich, SuchanaOchi, ShinichiroChaves, Maiana SilvaOchota, MałgorzataChavez, FranciscoOchsenkuehn, MichaelChecchi, VittorioOda, HirokiChekmarev, Sergei F.Oda, ShojiChemello, LilianaOdierna, GaetanoChen, Chih-ChiehOdintsova, N. A.Chen, Chun-JungOelkers, PeterChen, Chun-LiangOgasawara, ToruChen, GuoxunOgi, AsahiChen, Po-WenOgino, YoichiroChen, Sean Chun ChangOh, JunseoChen, ShukunOhba, SeigoChen, WeiOhno, HitoshiChen, Wen-ChingOhshiro, TakashiChenais, BenoitOhtsuka, TakashiCheng, JieOk-Seon, KwonCheng, Juei-tangOkuda, KazuhideCheng, LiangOkui, TasukuCheng, ZishuoOlcum, MelisCherouveim, EvgeniaOlczyk, KrystynaChevalier, FrançoisOlejnicka, BeataChi, Celestine N.Olimpi, ElissaChia, Jean-SanOlivares, Barlin O.Chiabrando, Gustavo AlbertoOliveira, RaquelChiang, Yi-HungOliver, Miquel PlanasChiapella, JorgeOlson, MichaelChiara, Nestola Maria GiuseppinaOmar, Syed HarisChico, José RuizO’neill, FrancisChiefari, EusebioOnisor, FlorinChiesa, MattiaOnukwufor, John O.Chilian, WilliamOpatovsky, ItaiChimienti, Guglielmina AlessandraOrd, KeithChin, LikangOren, MatanChinegwundoh, FrancisOrkoula, MalvinaChing, Lew LeeOrsini, GiovannaChitale, ShalakaOrtega, Miguel A.Chitrala, Kumaraswamy NaiduOrtega, Octavio Alonso CastelanChiu, Ing-MingOrtega, Roberto CoriaChiurazzi, PietroOrtiz, JorgeChlebek, DariaOscorbin, IgorCho, KyoungJooO’Shaughnessy, DouglasChoi, Jong-ilOsman, ChristofChoi, Soo-KyoungOstrowski, LaurenChoma, MichalOstrowski, RobertChong, RogerOstuni, MarianoChoquet, RémiOszako, TomaszChorianopoulou, StylianiOtsuki, MasayukiChoubey, SandeepOtsuki, TakemiChoudhury, Swarup RoyOtulak, KatarzynaChrist, BrunoOtulak-Kozieł, KatarzynaChristides, TatianaOuergui, IbrahimChristopoulos, PetrosOulhen, NathalieChristopoulou, AnastasiaOuro, AlbertoChrysanthakopoulos, Nikolaos A.Ozaki, ToshinoriChryssanthopoulos, CostasOzalp, CengizChtourou, HamdiOzimec, SinišaChua, Eng GuanPacak, ChristinaChung, Byung MinPacia, Marta Z.Chung, Tae NyoungPacifici, AndreaChyan, Chia-LinPacini, NicCiach, MichałPaciolla, CostantinoCiani, FrancescaPaczesny, ŁukaszCiani, FrancescoPaczos-Grzeda, EdytaCiasca, GabrielePadgett, PaigeCicero, ArrigoPagán, IsraelCiecierska, MartaPagano, DuilioCiemerych-Litwinienko, AnnaPagano, MarcelaCikurel, Eng. HaimPagano, StefanoCilia, GiovanniPage, CathieCiliberti, Maria GiovannaPage, MalcolmCimmino, GiovanniPaik, TaejongÇinar, ÖzgürPain, MatthewCipakova, IngridPaino, Eduardo NotivolĆirić, IvanPainter, Stuart C.Claringbould, AnniquePal, AjayClark, Nicholas J.Paladino, SimonaClarkson, Becky D.Palagani, AjayClemens, RobPalini, SimoneClemente, Filipe ManuelPalinski, RachelClemente-Suarez, Vincente JavierPallavicini, GianmarcoCodella, RobertoPalleschi, GiovanniCoelho, Antonio Victor CamposPalmieri, ErikaCohen, Ethan DavidPalomba, LetiziaColasanti, TaniaPalumbo, CarlaColeine, ClaudiaPamarthy, SahithiColin, CarolePan, Debra H.Collareta, AlbertoPan, YijunCollazo, Jaime A.Panarello, GiuseppeCollymore, ChereenPandey, AshutoshColquitt, Bradley M.Pandey, Nitin KumarColtell, OscarPandey, SheoComi, CristoforoPangestu, MulyotoComte, KatiaPanikov, NicolaiConflitti, PaoloPanitsa, MariaCongedo, PierluigiPankotai, TiborConn, Jan E.Panneerdoss, SubbarayaluConte, FrancescaPanpipat, WorawanConti, ValeriaPant, SameerContreras, OsvaldoPanter, Stephen NeilCook, Corey L.Paolini, AlessioCook, GerickePapa, VeronicaCooks, TomerPapadimas, George KonstantinosCooper, Arthur J. L.Papadimitriou, KonstantinosCoratella, GiuseppePapakonstantinou, EvgeniaCordani, NicolettaPapakostas, SpirosCornish, StephenPaparella, MartinCoro, GianpaoloPaquin-Proulx, DominicCorreia, InêsParameswaran, JananiCorso, GiovanniParashar, VijayCorte-Real, AnaPardos, Elena MainerCosoveanu, AndreeaParé, J. R. JocelynCosta, ElisabetePark, Chang-haCosta, ErnanePark, HyunCosta, Maria JoãoPark, JongsunCosta-Nunes, João PedroParker, DanCosta-Pinto, Ana RitaParmentier, EricCourdavault, VincentParrino, VincenzoCourtney, DavidParthasarathy, AnutthamanCourty, Pierre-EmmanuelParvez, Khan MohdCovo, ShayPasternak, Taras P.Cram, Erin J.Pastorino, PaoloCreamer, RebeccaPasztaleniec, AgnieszkaCretoiu, DragosPatel, ArshCrichton, Robert R.Patel, BrijeshkumarCristache, Corina MarilenaPatel, DevenCrosetti, ErikaPaterson, ClaireCroteau, DeborahPatil, Abhinandan SurgondaCruz, JoanaPatil, VeerupaxagoudaCsiszár, JolánPatkowska, ElżbietaCuenca-Martínez, FerranPatnaik, SouravCui, FengPatrício, PaulaCui, HaitaoPatterson, CarsonCui, LeqiPatz, SaschaCui, RongfengPaudel, AtmikaCull, BenjaminPaulose, Jiffin K.Cunha, EugeniaPaulraj, Raja SinghCunningham, Christopher W.Pavičin, Ivana SavićCurcic, MarijanaPaw, MilenaCuric, GoranPearce, NorrieCurtis, MarionPecci-Lloret, Miguel R.Cusato, JessicaPecka-Kielb, EwaCuypers, BartPedeux, RémyCyganek, LukasPei, Kurtis Jai-ChyiCzepczor-Bernat, KamilaPei, ShaopengCzirják, GáborPeitsidis, PanagiotisCzortek, PatrykPellegrini, MarikaDa Silva Carneiro, Sueli CoelhoPellegrini, SandraDa Silva Pontes, MontcharlesPeneva, VladaDa Silva, Liliana Machado R.RibeiroPeng, Weng KungDacheux, Jean-LouisPennati, FrancescaD’Addabbo, TrifonePensabene, VirginiaDai, Yunxiang (Sean)Pepe, FrancescoD’Alessandro, MirianaPepe, TizianaDall’Aglio, CeciliaPereira, FilipeDallaire, AlexandraPereira, Guilherme LuisDamaševičius, RobertasPereira, PatríciaDamerau, AlexandraPereira, Tiago JoséDande, Ranadheer R.Perek, BartłomiejDandekar, AdityaPere-Martinez, SilvinaDanish, SubhanPerestrelo, RosaDankel, ScottPérez, Ana PastorDar, ShowkatPérez, Jonathan H.Darling-Reed, Selina F.Pérez, Vicente RomoDas, SubhaPérez, Víctor QuesadaD’Ascenzi, CarloPérez-Donoso, Jose ManuelDaskalakis, KosmasPérez-Përez, GuillermoDaub, MatthiasPeriasamy, RameshD’auria, ValeriaPeron, GregorioDauth, StephaniePeroni, EdoardoDauvin, Jean-ClaudePerpetuini, DavidDavidson-Hunt, IainPerri, AnnaDavies, MichaelPerrino, Enrico VitoDay, Regina M.Perumal, SampathDe Araujo, Heloisa Sobreiro Selistre Pessoa, Rodrigo SávioDe Armas-Rillo, Laura Petca, RǎzvanDe Bellis, Luigi Peters, ThomasDe Carvalho Felippini, Ana LuizaPeterson, CraigDe Carvalho, Gleidson Giordano Pinto Petillon, JulienDe Cerio, Oihane Diaz Petow, StefanieDe Chiara, Francesco Petr, KotlíkDe Falco, Maria Pétriacq, Pierrede la Cruz-Márquez, Juan Carlos Petrie-Hanson, LoraDe La Serrana, Daniel Garcia Petritsch, BernhardDe La Varga, Herminia Petrosyan, Varosde León, Federico Abel Ponce Petrullo, LaurenDe Luca, Paola Petti, CarloalbertoDe Luca, Pasquale Pfeffer, Lawrence M.De Luca, Paula Mello Philibert, DanielleDe Lurdes Inácio, MariaPhillips, JadeDe Matos Macchi, Barbarella Picarella, Maurizio EneaDe Maturana, Evangelina López Pickard, AngelaDe Melis, Mirko Pickens, JeremyDe Menezes, Marcio Argollo Piecuch, AgataDe Miglio, Maria Rosaria Pierzchała-Koziec, KrystynaDe Molon, Rafael Scaf Piesik, DariuszDe Oliveira, Amanda Almeida Pilc, AndrzejDe Paula Baptista, RodrigoPiłsyk, SebastianDe Sanctis, Francesco Pilu, RobertoDe Silly, Romain Vuillefroy Pimentel, GustavoDe Sousa-Coelho, Ana LuísaPinart, ElisabethDe Souza, Danival José Pinello, Kátia CristinaDea-Ayuela, María AuxiliadoraPinto, SandraDebbaut, CharlottePintori, NicholasDebiton, ClémentPiomboni, PaolaDechaume-Moncharmont, François-XavierPiscopo, MarinaDel Gallo Di Roccagiovine, Maria MaddalenaPistorio, Valeriadel Gallo, Mddalena Pisu, Maria Carmeladel Giudice, AndreaPitarch, Aidadel mar Ramírez Fernández, MaríaPittet, Florentdel Refugio Castañeda-Chávez, MaríaPiva, Terrencedela Cruz, Thomas Edison Plaza, José Francisco SeguraDelcourt, MatthieuPlotkin, Lilian I.Delgado-Bermúdez, AriadnaPobiega, KatarzynaDelgado-Calle, JesusPoddighe, DimitriDellis, OlivierPogrmic-Majkic, KristinaDelorme, VincentPoidatz, JulietteDeniaud, AurélienPoirot, MarcDenisow, BozenaPokorski, MieczyslawDeochand, DineshPolak-Śliwińska, MagdalenaD’Errico, GiadaPolańczyk, AndrzejDeschenes, MichaelPolano, MaurizioDestexhe, AlainPoli, MauraDevigili, AlessandroPolicar, TomasDey, NandiniPolly, PatsieDey, Shyam SundarPoma, AdolfoDey, SupravatPombo, AnaDhanasiri, AnushaPonchel, FrédériqueDi Blasio, LauraPontoriero, AntonioDi Lorenzo, FrancescoPopova, AnetaDi Marzio, PieraPorcelli, SimoneDi Nardo, DarioPorras, AlmudenaDi Nora, Concetta Porta, Matteo DellaDi Pino, AntoninoPortela-Bens, SilviaDi Stefano, Barbara Posautz, AnnikaDi Tommaso, Morena Possenti, LucaDi Virgilio, Francisco Poston, BrachDi, RanPotaczek, Daniel P.Díaz-Flores, LucioPoulianiti, KonstantinaDiaz-Lanza, Ana MariaPoulicek, Mathieu E. A.Dibwe, Dya FitaPoupot, RemyDickmeis, ChristinaPozzi, CarloDidangelos, TriantafyllosPrabhakar, AditiDieci, GiorgioPrabhu, Antony HeroldDierickx, ErinPrasad, ManuDietrich, Christopher H.Prashanth, SuravajhalaDiharce, JulienPress, Charles McLeanDiMizio, GiulioPrezoto, Benedito C.Dinischiotu, AncaPrié, VincentDionyssiou, DimitriosPrlic, Jelena KoracDityatev, AlexanderProdromos, ChadwickDivashuk, MikhailProkic, MarkoDixit, AjayPryczynicz, AnnaDixit, NaveenPshenichnyuk, StanislavDkhar, Hedwin KitdorlangPsomopoulos, Fotis E.Dłużniewska, NataliaPszczółkowska, AgnieszkaDo Amaral, Danilo Trabudo Puitel, Adrian CǎtǎlinDo Paço, Ana Purudappa, Prabhudev PrasadDobrzańska, JoannaPusch, StefanDocea, Anca OanaPutman, NathanDodd, Richard S.Putz, PeterDoddamani, RajivPutzu, LorenzoDöffinger, RainerPuzio, IwonaDoley, DavidPyszko, PetrDoligalska, MariaQu, NingDolivo, DavidQuackenbush, SandraDominguez, Eddie G.Quagliariello, VincenzoDomínguez, Fadia Victoria CervantesQuaini, FedericoDomínguez, JoseQuattro, Joseph M.Domínguez, RaulQueinnec, EricDomínguez-Álvarez, EnriqueQueiros, JoãoDominguez-Vías, GermanQuerner, PascalDomsch, KatrinQuero, GraziaDonadel, GiuliaQuick, Quincy A.Donadu, MatthewQuiroga, Lorena BeatrizDonaire, LiviaQuiros, Carolina SitgesDonati, BenedettaRab, AndrásDonati, GiacomoRabaglio, ManuelaDong, FangcongRabbani, GulamDonnini, SandraRabchevsky, AlexanderD’Onofrio, MaraRachel, GilroyDonti, OlyviaRadenkovs, VitalijsDonze-Reiner, Teresa J.Radosavljević, TatjanaDorado, ChristinaRadošević-Stašić, BiserkaDorea, CaetanoRadoslavov, GeorgiDorn, MarcioRady, MostafaDory, DanielRaggi, CarlaDos Santos Nascimento, Luana Beatriz Ragusa, Maria AntoniettaDotsev, ArsenRagusa, SalvatoreDouglass, JanRahimi, FaridDougnon, GodfriedRahman, AsadurDouma, MountasserRahman, M. AzizurDoyle-Baker, PatriciaRahmani, RediDrakos, EliasRai, NavneetDresp-Langley, BirgittaRaia, TizianaDu, HuilongRaimundo, JoanaDu, QianRainho, AnaDuarte, AidaRajaguru, GulasekaranDubey, SushilRajamohan, ArunDuchateau, Marie JoséRajaure, ManojDuda, Marcel MateiRajput, SandeepDuderstadt, KarlRajput, VishnuDudhipala, NarendarRajput, Vishnu D.Dufossé, LaurentRam, Angia Sriram PradeepDukić, WalterRamachandran, RahulDumoux, MaudRamalhosa, Maria JoãoDunphy, Michael J.Ramalingam, SundarDuralija, BorisRamos, PatricioDuranti, ClaudiaRampanelli, ElenaDuranti, GuglielmoRandhawa, ImtiazDuregon, FedericaRanzato, EliaDurian, GuidoRaquel, LamaDurkalec-Michalski, KrzysztofRascio, AgataDutour, OlivierRasheed, FahadDutt, Taru S.Rashidieh, BehnamDutta, SudipRasmussen, SkyeDuvigneau, J. CatharinaRaspor, MartinDvoretsky, Alexander G.Ratajczak, MariuzDvoretsky, VladimirRatajczak, MonikaDwivedi, GarimaRatet, PascalDyche, JeffRather, IrfanDziekońska, AnnaRathinasabapathy, AnandharajanDziubek, KamilRaupach, AnnikaDzmitruk, VolhaRauseo, JasminEamens, Andrew L.Rauso, RaffaeleEbihara, LisaRavagnan, GiampietroEchauri, Sergio GarciaRaven, John AlbertEdae, Erena A.Rawski, JonathanEdit, FarkasRay, SamriddhaEdwards, LindseyRayan, AnwarEdwards, StellaRaza, Sayed Haidar AbbasEedara, BasanthRazvan-Cosmin, PetcaEfremenkova, Olga V.Reber, Arthur S.Egea, Mariana BuraneloRebolledo, Álvaro Efrén DomínguezEgger, BorisReboreda, AntonioEgger, JanReddy, Chandan G.Ehmann, RosinaRehman, AbdulEisenhaber, FrankRehrig, ErinEl Zowalaty, Ahmed Ezat Reis, FlávioElaswad, AhmedReix, NathalieElchaninov, AndreyRejinold, SanojElias, Maria CarolinaRekawiecki, RobertEljaafari, AssiaRendina-Ruedy, ElizabethElli, MarinaReybrouck, MarkEllis, Thomas Henry NoelReychler, HervéElliser, Cindy R.Ribeiro, HelenaEl-Mesery, MohamedRibeiro, TiagoElmoselhi, AdelRibeiro, Viviana PintoElnakib, AhmedRicci, SaraElsayed, MansourRicciardi, MariaElshafie, Hazem S.Ricciardi, Maria RosariaElsharkawy, MohsenRiccò, MatteoElsherbiny, NehalRichards, SeanElsner, MatthiasRichter, LukaszElumalai, PunniyakottiRichter-Dennerlein, RicardaElwan, HamadaRico, J EduardoEnany, ShymaaRiegert, JanEnciso-Martinez, A.Riego-Ruiz, LinaEndo, AkiraRiemann, DagmarEndo, KazuyoshiRiemma, GaetanoEndo, MakotoRiethmüller, ChristophEngel, NadjaRinaldi, CarmenEngl, TobiasRincón-Sánchez, Ana RosaEnguita, Francisco J.Rippa, SoniaEnko, DietmarRistić-Medić, DanijelaEnomoto, HirayukiRius-Pérez, SergioEnríquez, SusanaRivadeneyra-Domínguez, EduardoEpelde, FranciscoRizcallah, EdmondErb, CarlRizzarelli, EnricoErickson, TimothyRizzo, CarmenErismis, Ugur CengizRo, HyunjuEscoffre, Jean-MichelRobas, MarinaEscriu, FernandoRoberts, Catherine C.H. GutmannEscudé, ChristopheRobertson, JamesEscudero, OlgaRobey, Pamela GehronEsperon-Rodriguez, ManuelRobic, AnnieEsposito, GennaroRoca, JordiEsposito, GiuseppeRoccaro, AldoEsposito, LuigiRoche, EnriqueEsteban, Ma. AngelesRodrigo, Juan P.Evani, Shankar JaikishanRodrigo, LuisEvilia, CarynRodrigues, Andreia C. M.Exbrayat, Jean-MarieRodrigues, Pedro MiguelFaggiano, AndreaRodríguez, Ana MarchenaFaivre-Rampant, PatriciaRodríguez, Lourdes GudeFaix, James D.Rodríguez-Franco, AntonioFalguières, ThomasRodríguez-García, AlbaFalkinham III, Joseph O.Rodríguez-Reséndiz, JuvenalFanciulli, GiuseppeRogers, HilaryFanfarillo, EmanueleRogulj, Ana AndabakFanourakis, DimitriosRoisenberg, MauroFansa, HishamRojo, Maria AngelesFarì, GiacomoRokicka-Konieczna, PaulinaFarias, Jorge GRomano, DonatoFarina, LorenzoRomano, GiovannaFassl, AnneRomano, Giovanni LucaFassouane, AzizRomano, MarcoFatiukha, AndriiRomano, MaurizioFaure, SébastienRomano, NicholasFaustini, MassimoRoma-Rodrigues, CatarinaFazio, EsterinaRomera, ElenaFazio, FrancescoRoot, DouglasFecka, IzabelaRoot-Bernstein, MeredithFélix, Luís M.Ropelewska, EwaFelpeto, Aldo BarreiroRosenfeld, EliFeng, Jia-YihRosero-Garcia, DorisFeola, AlessandroRoss, Ian L.Feraru, ElenaRoss, MayaFerguson, Matthew L.Rossi, SimoneFernandes, Eto SilasRossiter, PaulFernandes, FábioRotherham, Ian D.Fernando, FerreiraRoudier, EmilieFerrante, AntonellaRouhafzay, GhazalFerrari, RobertaRoy, AmitFerrario, JasmineRoy, DipankarFerraz-de-Souza, BrunoRuban, DmitryFerreira, FernandoRuchin, AlexanderFerreira, FranciscoRueda-Ruzafa, LolaFerreira, Maria TeresaRuffin, FeliciaFerreira, NelsonRuggiero, GennaroFerreira, RodrigoRugman-Jones, Paul F.Ferrer, GerardoRuiz, HéctorFiallo-Olivé, ElviraRuiz, Rafael GinesFialová, AnnaRuiz-Albarrán, MiguelFichera, EpifanioRuiz-Jarabo, IgnacioFieber, Lynne A.Rumbos, ChristosFiedler, KonradRupnik, Marjan SlakFigueiredo, Bruno R. S.Russell, AlistairFigueiredo, Neusa L. L.Russini, ValeriaFijałkowski, KarolRusso, IsabellaFilice, MariacristinaRusu, DianaFilipovic, MiodragRuzafa, NoeliaFindlay, AmyRuzzier, EnricoFine, MichaelRybakowska, IwonaFinelli, CarmineRzymowska, JolantaFineschi, VittorioSaarela, MirkaFinimundy, Tiane C.Sabag, AngeloFink, DieterSabharwal, PriyankaFionda, BrunoSabino, MarcellaFischer-Friedrich, ElisabethSabri, FirouzehFišer, CeneSacchi, SilviaFjeldbo, Christina SætenSacher, EdwardFlaherty, KevinSachio, TsuchidaFlisikowski, KrzysztofSadasivam, MohanrajFocosi, DanieleSadhukhan, PritamFogacci, FedericaSaha, NirmalyaFolch-Mallol, Jorge L.Sahgal, PranshuFolco, Hernan DiegoSaint-Pol, JulienFonseca-Alves, Carlos EduardoSaito, JumpeiForero-Castro, MaribelSaitoh, KenjiForero-Quintero, Linda S.Sakai, AtsushiForest, FabienSakai, MasahiroFornasiero, EugenioSakakibara, HiroyukiForte, PedroSakanyan, VeharyFossa, PaolaSakata, MasahiroFoti, GiandomenicoSakata, NaoakiFotouhi, OmidSako, KaoriFozzatti, LauraSakuma, Rebeca KawaharaFrachon, LéaSala, LucaFrączyk, TomaszSalafia, FabioFraley, Gregory S.Salagre, EstelaFrancesca, RappaSalegui, Ana Belén FernándezFranceschetti, LorenzoSalehzadeh-Yazdi, AliFranceschi, PieroSalinas, ValeriaFrancesco, AnielloSalman, MootazFrancis, FredericSalomone, AlbertoFrancisco-Morcillo, JavierSalto-Gonzalez, RafaelFranco, Fernando FaríasSalvi, MassimoFrançois, CasasSambaraju, KishanFranczak, AnitaSammi, ShreeshFrank, Alexander HSamylina, Olga S.Frank, Erik T.Sánchez Del Pino, Manuel M. Fraschini, RobertaSánchez, Miguel Ángel SogorbFratini, FedericaSánchez-Gómez, RubénFrauenknecht, KatrinSánchez-Labrador, LuisFreen-van Heeren, JulianSánchez-Muñoz, LauraFreidin, Mona M.Saneyasu, TakaokiFreisinger, EvaSangha, JatinderFrey, AnnaSankararaman, SenthilkumarFrey, KathleenSano, TeruoFrick, ManfredSantacroce, LuigiFriemel-Bauersfeld, JulianeSantander, JonesFrietze, SethSantarpino, GiuseppeFrigo, LúcioSanti, MelissaFrilling, AndreaSantoro, AngelaFritz, JörgSantos, JerranFroyen, ErikSantos, João Miguel Marques DosFruhbeck, GemaSantos, José MariaFrustaci, Anna MariaSantos, Raquel De Cássia DosFruttero, Leonardo L.Santos, RégisFrýdlová, PetraSantos, RenatoFthenakis, GeorgeSantosh, K. C.Fu, FuyouSantovito, GianfrancoFuehrer, Hans-PeterSanz, Adrián VarelaFuentes, Màrius V.Sarabian, CécileFujimori, KoSaran, UttaraFujimura, ShintaroSaratale, Rijuta GaneshFujisaki, HiroshiSaravanakumar, KandasamyFujita, MasayukiSarkar, PoulamiFukuda, RyuichiSarker, Mostafa KamalFukushima, AtsushiSarkozy, MartaFung, Sai-fuSarma, PranjalFurdui, BiancaSarode, Gaurav V.Furtak, KarolinaSarr, DembaFuruita, HirofumiSarropoulou, ElenaFusco, AndreaSartini, FrancescoFuss, TheodoraSartoretto, StephaneGabrić, DraganaSartori, Andrea M.Gabrielson, EdwardSarul, MichałGadermayr, MichaelSasagawa, MasaGagniuc, PaulSasso, Gregorio DalGaihre, BipinSato, EiichiGaitán-Cepeda, Luis AlbertoSatta, YokoGalicka, AnnaSaul, DominikGalindo, Fernando ShintateSavoca, SerenaGalindo-Riaño, DoloresSavvateeva-Popova, E. V.Gallardo, LeonorSayan, Deb DuttaGallo, CarmelaScaccini, DavideGallo, GiovanniScacco, SalvatoreGaman, Mihnea-AlexandruScavo, AurelioGambichler, ThiloScavuzzo, Carlos M.Gambús, María ArreguiSchäfer, JanaGamez, CristinaScharf, AnneGammazza, Antonella MarinoScharf, InonGamsjaeger, SonjaSchewe, MatthiasGandla, Madhavi LathaSchlechter, Rudolf O.Ganguly, AnindyaSchloot, Nanette C.Gao, TianxiangSchlotzer-Schrehardt, UrsulaGarbayo, InésSchmidt, MarcinGarcia, Angel Arturo GuevaraSchmidt, MaximilianGarcia, Brandon L.Schmidt, Michael RahbekGarcia, Cristina MenizSchmitz, IngoGarcía, Leonardo Azael GarcíaSchmoll, DieterGarcia, MiriamSchnabel, UtaGarcía, Vicente GonzálezSchnyder, ManuelaGarcía-Delpech, SalvadorSchoon, AdeeGarcia-Descalzo, LauraSchrama, DeniseGarcía-Gasca, Silvia AlejandraSchreiber, RainerGarcía-Pola, María JoséSchröder, AgnesGarcía-Rodríguez, NéstorSchröder, SvenGarcía-Sánchez, SusanaSchroer, SibylleGarcía-Soidán, Jose L.Schuberth, Hans J.Garcovich, SimoneSchwarz, MónicaGardiman, MassimoSchwarz, PatrickGargiulo, PaoloSchwind, SebastianGariglio, MarisaScibior, AgnieszkaGarrido, ClaudiaScioli, Maria GiovannaGartner, MariekeScioscia, CrescenzioGasmi, LeilaScolieri, PalmaGaspar, HugoScott, CatherineGasparski, AlexanderSeal, BruceGast, Thomas J.Sébastien, CouetteGatti, MauroSee, Kay ChoongGatto, Bernardo B.See, Pao TheenGatto, EliaSeeliger, ClaudineGaudio, AgostinoSegatto, MarcoGaul, DavidSeibel, HenrikeGaustad, Ann HelenSeifert, StephanieGavert, NancySeixas, AnaGavish, MosheSekino, YoheiGaweł-Bęben, KatarzynaSelim, Abdelfattah M.Gawlińska, KingaSellegounder, DuraiGawroński, JacekSempio, CristinaGazzola, AndreaSenderowitz, HanochGecheva, GanaSentenska, LenkaGennari, RoccoSequeira, CarlosGentès, SophieSérandour, AurélienGentil, PauloSergi, Pier NicolaGenzor, PavolSermsathanasawadi, NuttawutGeorgel, PhilippeSeroussi, EyalGerbaud, EdouardSerra, Claudia R.Gerbino, AndreaSerrano, Maria VillaGerding, HeinrichSerrano-Brañas, Claudia InésGerdol, MarcoServín-Garcidueñas, Luis EduardoGershoni, MoranSette, MarcoGhalem, Bachir RahoSevilla-Morán, BeatrizGhanem, NasserShabardina, VictoriaGherman, Calin MirceaShabbir, WaheedGhiroldi, AndreaShafiq, AnumGhoreifi, AlirezaShafiq, SarfrazGhosh, MonojShahbaaz, MohdGhosh, SantoshShahida, BushraGhosh, SharmilaShakeri, MajidGhosh, SoumitaShamsaeI, BehrouzGiallauria, FrancescoShamsi, ShokoofehGianturco, LuigiShanmugam, MuruganandanGica, NicolaeShanmugam, Shankar GanapathiGienger, C. M.Shao, ChangweiGilbert, LeonaShapiro, Stuart Z.Giliomee, Johannes HumanSharakhova, MariaGilles, AndréSharma, GayatriGil-Santana, HélcioSharma, KavitaGiordano, AntonioSharma, ShrikantGiovanni, NicolettiShavit, JordanGiovenazzo, PierreShaw, PriyankaGiri, Sib SankarSheeran, LoriGiroux, VéroniqueShehata, MohamedGismondi, AngeloShekhovtsov, SergeyGiuffrè, Angelo MariaShen, JiangchuanGiulianini, Piero GiulioShen, JianqiangGiurg, MirosławShen, YangGkantidis, NikolaosSheng, YueGłąbska, DominikaSheppard, Mary N.Glick, Bernard R.Sheshadri, NamrathaGłowacka, KatarzynaSheteiwy, MohamedGnat, SebastianShewade, LeenaGodinho, CatarinaShi, De-LiGodos, JustynaShi, SonglinGokulan, Ravindran CaspaShi, ZhiGolla, Jaya PrakashShibata, HidekiGolubkina, NadezhdaShiina, MarisaGómez, Pedro A. JiménezShilts, TurksenGómez, Sonia AlejandraShima, YoshihitoGómez-Carmona, Carlos DavidShimaoka, MotomuGonçalves, Ana CristinaShimba, ShigekiGonçalves, João F.Shimizu, YujiGoncharov, AntonShinde, AparnaGong, GuanghuiShinoda, MasahiroGoñi, Guillermo ZalbaShort, Jeffrey W.González, AlbertoShoshan, MariaGonzález, José A.Shukla, KirtikarGonzález-Álvarez, Vicente HomeroShukla, Pushp SheelGonzalez-Parra, GilbertoShumilov, EvgeniiGórniak, DorotaSiadjeu, ChristianGorrell, MarkSiatkowski, IdziGospodarek, JaninaSiddique, Mohammad Abdul MominGostin, Irina NetaSiddiqui, ArifGötz, Klaus-PeterSidler, DanielGould, JohnSidorkiewicz, IwonaGoulet, Dennis R.Sieber, SimonGouveia, Élvio Rúbio QuintalSiebert, Hans-ChristianGowda, HarshaSiessere, SelmaGowthaman, RagulSignorini, CinziaGoyal, RamanSikalidis, AngelosGoyer, ClaudiaSillerud, Laurel O.Gozdowski, DariuszSilva, CatarinaGrabarczyk, DanielSilva, Daniela Joyce TrujilloGrabau, Larry J.Silva, Jerson L.Grabherr, SilkeSilva, Thiago VilarGrabinska, KarionaSilva, VanessaGrabińska-Sota, ElżbietaSilveira, Neidiquele M.Graczyk-Jarzynka, AgnieszkaSimbirtsev, AndreyGraf, ThomasSimenko, JozefGraham, Melanie L.Simigdala, NikianaGrammatikopoulou, Maria G.Simitzis, PanagiotisGramsbergen, JanSimm, StefanGranberg, DanSimões, PaulaGranica, SebastianSimon, LukeGrasman, JonathanSimon, MartinGrässel, SusanneSimons, MikaelGrassilli, EmanuellaSinger, Bernhard B.Gratl, AlexandraSinger, Stacy D.Gravili, CinziaSingh, Abhishek KGreco, NicolaSingh, AnandGreen, PhilSingh, GurdeepGregorini, MarilenaSingh, VikasGrieco, FrancescoSingh, Vivek KumarGrieco, ValeriaSinghal, Sandeep K.Griffith, MaySingla, BhupeshGriga, MiroslavSinsch, UlrichGrill, MagdalenaSinutok, SutineeGrimaldi, PiercarloSiqueira, NatalyGrimm, MarcusSistla, SrinivasGrimm, Marcus Otto WalterSitvarin, Michael I.Grimm, W. D.Sivaev, Igor B.Grippi, FrancescaSivakumar, SushamaGrishkan, IsabellaSivarajan, Sajeevan RadhaGrones, PeterSkartveit, JohnGroot, Hilde E.Škerl, Maja Ivana SmodišGrosse, RobertSkliros, DimitriosGrosshans, Claudine BleykastenSkoracki, MaciejGrote, KarstenSkorokhod, OleksiiGrotz, Travis E.Škorput, DubravkoGrover, SajjanSkrzypczak, Maciej IekGrubczak, KamilSladonja, BarbaraGrubov, VadimSlemr, FranzGründer, StefanSłomińska-Wojewódzka, MonikaGuarnaccia, VladimiroSlominski, AndrzejGuedes, SofiaSmaldone, GiorgioGuerrero, CarlosSmedile, FrancescoGuerriero, IlariaSmeller, LaszloGuevara, NataliaSmiley, Peter C.Guibert, ChristelleŚmiłowska, KatarzynaGuieu, RégisSmirnov, Alexey V.Guillaume, OlivierSmirnova, E. A.Guillen-Grima, FranciscoSmith, Adam R.Güldiken, NurdanSmith, ChristopherGültas, MehmetSmith, J. KellyGunaje, JayaramaSmykal, PetrGuo, BingSoares, PaulaGuo, LilongSobolev, DmitriGupta, Adarsh K.Soffritti, IreneGupta, KuldeepSolano, FranciscoGurusamy, RamanSoldo, AlenGusev, AlexanderSoldovieri, Maria VirginiaGustafsson, RasmusSoler, Alejandro RivasGuzzi, FrancescoSolovchenko, AlexeiHabas, KhaledSoltani, NourolahHabila, Mohamed A.Somasundaram, PreethiHackett, Perry B.Son, KeunbadaHackman, LavenderSong, QishiHaga, SatoshiSonowal, HimangshuHägerling, RenéSorg, OlivierHahne, JensSoria, Federico N.Haigh, AnthonySoriano, PilarHajdari, AvniSoriano-Úbeda, CristinaHakim, Md AbdulSorokin, Vitaly A.Hallam, StevenSosa, LilianHallerman, EricSosnowska, KatarzynaHalterman, DennisSotelo, Ana I.Halvatsiotis, Panagiotis G.Sousa, Antonio Alberto RodríguezHamaguchi, ToyohiroSousa, FilipaHamaoui, KarimSouto, Daniel GarcíaHamerly, TimothySovak, GuyHan, Dong-WookSowinska, NataliaHantusch, BrigitteSpagnuolo, GianricoHanus-Fajerska, Ewa JoannaSpanò, NunziacarlaHaq, IramSpartalis, MichaelHardt, JuliaSpeijer, DaveHarms, ChristophSpinelli, FrancescoHaro, GonzaloSpletter, MariaHarris, LarrySponsler, DouglasHarris, StephenSpratt, Donald E.Hart, SteveSpray, David C.Hartman, MariuszSprícigo, José Felipe WarmlingHartung, JensSpur, Bernd W.Harvey, InnocenceSridurga, MithraprabhuHasegawa, TakaakiSrivastava, AkritiHasipek, MetisStaderini, EdoardoHassan, Sherif T. S.Stancampiano, AugustoHassani, Mohamed-AmineStanciu, Gabriela-DumitritaHassanpour, AmirStanek-Tarkowska, JadwigaHata, MasakiStaniek, HalinaHathout, RaniaStanke, FraukeHatteland, Bjørn ArildStarfinger, UweHausladen, HansStarsich, FabianHayakawa, YokuStavi, IlanHayden, Benjamin Y.Stavraka, CharaHazawa, MasaharuSteigen, Sonja ErikssonHe, ChangSteinberger, YosefHe, MingyuSteinhagen, DieterHealey, AdamSteinheim, GeirHeckmann, StefanStephan, JonathanHefferon, Kathleen L.Ster, CélineHegde, BinduStevanovic, JevrosimaHeide, MichaelStoccoro, AndreaHeld, SteffenStöcker, FabianHeller, JanoschStoddard, ShanaHellgren, KimStoklosa, TomaszHelton, Rebekah R.Stöllberger, ClaudiaHenderson, GreggStrand, Michael R.Hendrik, BargelStröm, LenaHenein, ChristinStryamets, NatalieHenklewska, MartaSu, BaofengHensen, LucaSu, TaoHeo, JeongSuárez, JuanHer, Lu-ShiunSubbotin, Sergei A.Heragh, Esmaeil DoustkhahSubotički, TijanaHeraty, JohnSubramanian, SowmyalakshmiHerchenröder, OttmarSubramaniyan, BoopathiHernández-Martínez, PatriciaSuchý, JiříHernández-Urcera, JorgeSugino, ShigekazuHerr, IngridSukumari-Ramesh, SangeethaHerraiz, TomasSulek, AnnaHerranz-Martin, SaulSultan, Laith R.Herrero, María JesúsSun, Feng-HuaHerrick, Jason R.Sun, JianxinHerrin, DavidSun, Yung-ShinHess, Michael W.Sundaram, Sivaraj MohanaHesson, JennySuomalainen, MaaritHidari, KazuyaŠupljika, FilipHiebert, LaurelSurana, PriyankaHigashitani, AtsushiSurapaneni, Krishna MohanHiggins, JamesSurdacka, AnnaHilbert, FriederikeSutanto, HenryHildenbrand, GeorgSutton, KateHilke, Franz JoachimSuzuki, ArataHill, JacobSuzuki, ToshikazuHines, Dustin J.Svanvik, JoarHiraga, ToruSvicher, ValentinaHiroyoshi, SatoshiSwan, Pamela D.Ho, ChengyingSynakiewicz, AnnaHoang, Nam V.Szászi, KatalinHoehn, KyleSzeleszczuk, ŁukaszHoffmann, Lucia VieiraSzili-Kovács, TiborHoffmann, MarkusSztal, TamarHojski, AljazSzuki, MichioHolalu, Srinidhi V.Szyfter, KrzysztofHolert, JohannesTabata, RyoichiHolt, BillTabunoki, HirokoHolt, StephenTagliavia, MarcelloHomma, TakujiroTai, HelenHooda, JagmohanTaiar, RedhaHorgan, FinbarrTait, CheyenneHorn, IvoTakahashi, HidekiHorsburgh, K. AnnTakayuki, TsukuiHorst, E. H.Takebayashi, ShigeoHorvát, SzabolcsTakizawa, ShuheiHorvath, BalazsTalà, AdelfiaHoshide, Aaron K.Tamariz, ElisaHosokawa, NobukoTambaro, Francesco PaoloHossain, MotaherTamburino, RacheleHotos, George N.Tamkovich, SvetlanaHou, ShaopingTammaro, PaoloHou, Wen-ChiTamura, KeitaHoughton, Karen M.Tan, Bee K.Hoyer-Fender, SigridTan, Nguan SoonHsieh, Feng-ChiaTanabe, ShihoriHsu, Te-HuaTanalgo, Krizler CejuelaHsu, Tsung-ITanase, AlinaHsu, Yu-ChiaTang, AnnaHu, DongjianTang, FengRuHu, JiayingTanimizu, NaokiHuang, ChangwenTanwar, VineetaHuang, Guan-JhongTapia, Alejandro A.Huang, KunTarantino, GiovanniHuang, TaoTarasco, EustachioHuber, JörgTashima, ToshihikoHuché-Thélier, LydieTaszakowski, ArturHuchzermeyer, BernhardTavanti, FrancescoHudina, SandraTchetina, Elena V.Huff, AlyssaTebani, AbdellahHülsmann, MichaelTellgren-Roth, ChristianHumphreys, JohnTeraoka, HirokiHunter, DavidTesta, UgoHunter, ElizabethTheil, GeritHuppi, KonradTheopold, UlrichHurwitz, StephanieTheotokis, PaschalisHussain, MubsharTheuerkauf, JörnHwang, JeonghoThibault, DelphineHybiske, KevinThirunavukkarasu, ShyamalaHyde, Kevin D.Thiyagarajan, VengatesenHyun, Sang-HwanThomson, JackIafolla, MarcoThorenoor, NithyanandaIaria, CarmeloTian, Fang-BaoIbaba, Jacques DavyTian, SonghaiIbrahim, Mahrous AbdelbassetTiano, LucaIchinose, KatsuyaTien, An-ChiIentile, RiccardoTikhe, Chinmay V.Iglesias, Olaia MartinezTirosh-Levy, SharonIguchi, TaisenTisi, RenataIkeda, Kyojiro N.Tittarelli, AndrésIlias, IoannisTiwari, Shashi KantIlinykh, Philipp A.Tiwari, SudeepIlyinskii, Petr O.Tkach, NataliaIm, Seung-SoonTkaczuk, CezaryImai, KenichiroToczylowska-Maminska, RenataIñaki, GarmendiaTodorov, SvetoslavInfante, MarcoToennes, Stefan W.Infascelli, FedericoTogawa, ToruIniesta-Cuerda, MaríaTokita, YoshihitoInomura, KeisukeTokuda, EiichiIntrona, FrancescoTokuda, GakuIorizzo, MassimoTokuyama, TakeshiIqbal, Shahzad ZafarTomaszewska, EwaIrfan, MohammadTomkin, GeraldIrla, MartaTong, MingmingIrum, KhanToriba, TaiyoIrurtia, AlfredoTornel, Eduardo LarribaIsegawa, YujiTornero-Aguilera, Jose FranciscoIshii, Ken J.Toro, Mario DIsigonis, PanagiotisTorraco, AlessandraIslam, MonirulTorralva, Fidel GonzalezIsola, GaetanoTorres-Collado, LauraIsozaki, AkihiroTorres-Sanchez, SoniaIto, TadashiTorricelli, MartinaIwai, AtsushiToscani, DeniseIwanicki, AdamToševski, IvoIwata, AtsushiTosti, TomislavIzabela, Kern-ZdanowiczTotoń, EwaIzadi, HamzehTovoli, FrancescoIzawa, Kazuhiro P.Tozer, PeterIzzo, CarmineTragust, SimonJabbour, FlorianTran, Hung QuangJackson, Mark A.Tran, Hung-VuJacob, Harrys Kishore CharlesTraversari, SilviaJacob-Dubuisson, FrançoiseTremblay, Bénédicte L.Jacomin, Anne-ClaireTrenti, MassimilianoJagosz, BarbaraTrentin, AndreaJahan, Mohammad ShahTrentzsch, KatrinJain, PrashantTreviño, VictorJakobs-Schönwandt, DesireeTrewavas, Anthony J.Jakubska-Busse, AnnaTriantafyllidis, VassiliosJakus, ZoltanTribst, João Paulo MandesJallet, DenisTricarico, ElenaJalli, MarjaTrifan, AdrianaJamshidi, MohammadTripathi, Siddharth KaushalJanauer, Georg A.Tripathi, Vishnu P.Janigro, DamirTripon, FlorinJanosi, LorantTripp-Valdez, Miguel A.Janovska, PavlinaTruman, Benedict I.Janssens, MarcTsai, Kun-LingJarret, RobertTsai, Shih-JenJarzebski, MaciejTsakovska, IvankaJasińska, Anna KatarzynaTsaniklidis, GeorgiosJaszczur-Nowicki, JarosławTsantarliotou, Maria P.Javitch, Jonathan A.Tsaousis, Anastasios D.Jayachandran, MuthukumaranTsingotjidou, AnastasiaJaźwińska, AnnaTsironi, FannyJedrejek, DariuszTsoi, HoJeffs, AndrewTsuchiya, HiroyukiJeong, Jin SuTsuruda, ToshihiroJeong, Keun-YeongTucker, ErikaJerônimo, Gabriela TomasTufarelli, VincenzoJeucken, AikeTunon, HakanJevremovic, SladjanaTunuguntla, Hari Siva Gurunadha RaoJiang, BoTurco, Luigi CarloJiang, HuaTuriák, LillaJiang, Jian-PingTurner, MarkJiménez, Randall R.Tuttobene, Marisel RominaJimenez-Olmedo, Jose M.Tyteca, DanielJiménez-Ruiz, JesúsUchida, ShizukaJiří, PatokaUebanso, TakashiJo, Dong HyunUgarriza, Raquel AparicioJo, HyunbinUlasov, IlyaJobling, MalcolmUlrich, SebastianJockusch, Brigitte M.Ungureanu, CameliaJoerg, BrigitteUpdegrove, Taylor B.Joles, Jaap A.Uppulury, KarthikJong, Chian JuUras, Iris Z.Jordana, RafaelUrban, PhilippeJörg, GrosshansVadrucci, Maria RosariaJørgensen, Hans Jørgen LyngsVagni, MoniaJose, MarcoVagnozzi, RonaldJoshi, Abhayraj S.Vahdati, KouroshJoshi, GauravVaidotas, StankevičiusJoshi, SameerValcarcel, FelixJosse, ClaireVale, PauloJósvai, Júlia KatalinValencia, Alfredo TéllezJourdain, AlexisValente, Nicola A.Juan, Yung-ShunValentim, AnaJubeau, SébastienValenzuela, Pedro L.Juliusson, GunnarValenzuela, RodrigoJuncar, MihaiVallebona, Paola SinibaldiJung, KlausValletta, AlessioJung, Tae SungVallicelli, Elia ArturoJúnior, Osmindo Rodrigues PiresVallius, ElisaJunior, Valdir F. VeigaValm, Alex M.Jurisch-Yaksi, NathalieVamanu, EmanuelJuszczak, Grzegorz R.van Den Berg, Cássio Kabir, Mohammad FaujulVan Der Giezen, MarkKabisch, StefanVan Der Laan, MartinKaboli, Saeedvan der Nest, Magriet A. Kacaniova, Miroslavavan Dobben, Han Kacsoh, Balintvan Eldik, Rudi Kaczmarek, AgataVan Haute, LindseyKadeethum, TeeratornVan Hoek, Bart Kaden, ReneVan Schothorst, Evert M. Kaelber, JasonVan Wuytswinkel, Olivier Kaether, ChristophVanek, DanielKagiya, TadayoshiVanhee, CelineKain, VasundharaVannini, AndreaKaldhone, PravinVanwalscappel, BénédicteKalinin, Vladimir I.Vanzi, FrancescoKaltsatou, AntoniaVarela, Maria CarolinaKamble, Shyam H.Varela, SusanaKamel, SarahVargas, FélixKamolz, Lars P.Vargova, KarinaKamran, MohammadVaris, SailaKang, ChulHeeVarju, CeciliaKang, Woo HyunVarnon, Christopher A.Kano, KiyoshiVarra, MichelaKanzaki, NatsumiVarricchio, EttoreKao, Cheng-YenVarsaki, AthanasiaKapellos, George E.Varvara, GiuseppeKarakavuk, MuhammetVasaikar, Suhas V.Karakawa, SachiseVasaitis, RimvysKarakostis, AlexandrosVasamsetti, Balamurali KrishnaKaran, RamVasileiadis, IoannisKaranastasi, EiriniVasos, PaulKaranis, PanagiotisVassiliou, Alice GeorgiaKarimi, ElhamVassilopoulou, EmiliaKarimzadeh, SedigheVatca, SorinKarkanis, AnestisVatopoulos, AlkiviadisKarlović, ZoranVaz De Carvalho, CarlosKarnati, Konda ReddyVaz-Moreira, IvoneKarpiński, Tomasz M.Vázquez-de-Aldana, Beatriz R.Karreman, Matthia A.Vazquez-Nunez, EdgarKarwowska, MałgorzataVécsei, LászlóKashif, MuhammadVelazquez, MiguelKasperski, AndrzejVelena, AstridaKatinakis, PanagiotisVelot, EmilieKato, HideoVencl, Fredric V.Kato, TakafumiVendl, TomasKato, TakamitsuVenieraki, AnastasiaKatsafadou, AngelikiVenisse, NicolasKatsani, KaterinaVenkatachalam, MekalaKatsigiannis, StamosVerberk, WilcoKatz, OfirVerde, FrancescoKauffmann, KiraVergara, DanieleKaur, AnisaVerhoeven, AdrieKavaliauskas, PovilasVerma, VikashKavran, MihaelaVeronico, PasquaKawai, TatsuoVersaci, MarioKawakami, MasanoriVerticchio, AliceKawamura, TakahisaVerwaal, Victor JilbertKayzer, DariuszVeskoukis, Aristidis S.Kazanidis, GeorgiosVest, Katherine E.Kazlauskas, ArunasVestergaard, Lau K.Ke, Po-YuanVeyron-Churlet, RomainKeasling, Jay D.Vezina, Chad M.Kędzierska-Mieszkowska, SabinaVicente-Manzanares, MiguelKeeler, JasonViciedo, Dilier OliveraKeflie, Tibebeselassie SeyoumVictorio, Carla Bianca LuenaKeiffer, Timothy R.Vidal, MiguelKeiner, MichaelVidal, TâniaKelarakis, AntoniosViegas, CarlosKenzaka, TsuneakiVieira, JorgeKeogh, KateVieira, Mônica LarucciKeshavarz, MeysamVigliotta, GiovanniKessi-Perez, EduardoVijayakumar, SekarKhan, AbbasVila, Alberto MolaresKhan, IrfanVilla, ChiaraKhan, Mohammad ImranVillanueva, Javier RodríguezKhan, ShahanshahVincent, AmyKhanal, Bishnu PrasadVinha, AnaKhapugin, Anatoliy A.Vinogradov, MarkelKhashayar, PatriciaVisconti, LucaKhemmar, RedouaneVisentin, AndreaKheradpezhouh, EhsanVitali, AndreaKhotimchenko, Maxim Y.Vizzarri, FrancescoKhrapko, KonstantinVlahos, NikolaosKieliszek, MarekVlainić, JosipaKienzle, ArneVogel, JohannesKijpornyongpan, TeeratasVolcho, KonstantinKim, Chang-BaeVolk, Andrew G.Kim, ChunkiVoloshin, ArkadyKim, Ho BangVolpert, VitalyKim, Hoonvon Eyben, Finn EdlerKim, Jaeyoonvon Fragstein, PeterKim, Ki Taevon Oheimb, Parm Viktor Kim, Ki-Hongvon Strandmann, Elke Pogge Kim, Ki-YoungVotypka, JanKim, Kyung-MinVrentas, Catherine E.Kim, ManuelaVukicevich, EricKim, Min JungVukotić, GoranKim, MinjooVurukonda, Sai Shiva Krishna PrasadKim, Peter W.Wadham, CarolKim, Ryong NamWagensommer, Robert PhilippKim, Seonghun HoonWagner, HerbertKim, SunghanWaiker, PrashantKim, Sung-HoonWakiguchi, HiroyukiKim, Sung-KyoWalker, Douglas G.Kim, Tae KonWalker, LinKimura, Masahito T.Walocha, JerzyKinnby, AlexandraWang, BoKino, TabitoWang, Ching-WeiKirubakaran, PalaniWang, DataoKische, HannaWang, GuannanKishimoto, ShinjiWang, HsiuyingKitagawa, SeiichiWang, HuafengKitowski, IgnacyWang, JianxuKiyanagi, YoshiakiWang, JunlingKizheva, YoanaWang, LihuaKlaoudatos, DimitriosWang, Tong-HongKlarić, Maja ŠegvićWang, Wen-DerKlaus, SusanneWang, WenhuiKleczka, AnnaWang, XiaoliangKleczkowska, PatrycjaWang, XishaKleszcz, RobertWang, YafeiKlewicka, ElzbietaWang, Yang-KaoKlontzas, MichailWang, ZhenKmita, MarieWang, ZhenghanKnelson, Erik H.Wang, ZhoufeiKnobloch, KarstenWanrooij, SjoerdKnoll, MarkoWard, LeighKnox, MatthewWarzecha, ZygmuntKnudsen, Thomas B.Wasilewska, EwaKoba, TakatoWasniewska, MalgorzataKoblmüller, StephanWasylka, Justyna PłotkaKobza, RichardWatanabe, ShigeruKoch, FranziskaWatling, LesKochanowski, MaciejWaxman, JoshuaKochat, VeenaWeber, Donald C.Koga, FumitakaWeeks, KateKoga, HironoriWegrzyn, AlicjaKogawa, TakahiroWegrzyn, GrzegorzKöhler, AlmutWęgrzyn, GrzegorzKohn, Julie C.Wehling, PeterKok, Philippe J. R.Wehrend, AxelKokokiris, LambrosWei, LijunKokoszyński, DariuszWein, AlexanderKoksharova, Olga A.Weiser, Douglas C.Kolbe, DianaWekwejt, MarcinKolesnikov, GennadyWende, Adam R.Kolukisaoglu, ÜnerWerbrouck, Stefaan P. O.Komaravolu, Ravi KumarWerner, EnriqueKomatsu, DavidWernly, BernhardKomiya, ErikoWeßels, IngaKompotis, KonstantinosWhite, ChristineKonantz, MartinaWhite, JamesKong, WeilongWhitford, RyanKonopińska, JoannaWhittington, EmmaKonopka, TomaszWia̧cek, Agnieszka EwaKontakiotis, GeorgeWichmann, GunnarKontos, Christos K.Wickersham, TryonKonwerski, SzymonWięcek, MagdalenaKool, JeroenWiegard, MechthildKooyman, Frans N. J.Wiegertjes, Geert FritsKoozehchian, MajidWielstra, BenKoren, AmnonWierzbowska, JadwigaKornilov, SergeyWijesekera, ThiliniKorona-Glowniak, IzabelaWilczyński, Jacek R.Korytář, TomášWillcox, CarrieKoschella, AndreasWillems, Mark Elisabeth TheodorusKosić, Ivana VitasovićWilliams, Paul F.Kosik-Bogacka, DanutaWilliner, VerónicaKosinsky, Robyn LauraWilson, RodKostrakiewicz-Gierałt, KingaWisniewski, MarekKostyushev, DmitryWitte, Hanno M.Kotcon, James B.Wlaź, PiotrKotteda, V. M. KrushnaraoWłodarczyk-Stasiak, MarzenaKotula-Balak, MałgorzataWłoga, DorotaKou, XiaoxingWójcik-Gront, ElżbietaKouokam, Joseph CalvinWojszel, Zyta BeataKouril, RomanWolf, MatthiasKoutelidakis, Antonios E.Wolf, ThomasKoval, MichaelWollebo, Hassen S.Kowal, PrzemyslawWolny, TomaszKowalska, KarolinaWong, Duo Wai-ChiKowalski, ThayneWong, Juliet M.Kozak, AnnaWong, LeeKozaki, AkikoWong, TentsaoKozdrój, JacekWood, AndrewKozieł, EdmundWorni, MathiasKozłowska, MariolaWoroniecka, KarolinaKozlowski, Michael R.Wöstemeyer, JohannesKrajewski, PawelWozniak-Knopp, GordanaKrakowski, LeszekWróbel, TomaszKraljevic, SonjaWu, Chun-ChiKrams, IndrikisWu, HannahKranz, StefanWu, Li-HsinKraska, ThorstenWu, RuoxiKravats, AndreaWyszkowska, JadwigaKrazinski, BartlomiejXhetani, MeritaKreft, IvanXi, LinKreslavski, VladimirXia, ‪ShengqianKrieghoff-Henning, EvaXie, ShengkunKrier, FrançoisXing, WeiKrisch, JuditXiong, JiaKrishnasamy, GopinathXu, ChengqiKristensen, GunnarXu, JunKristo, AleksandraXu, RongKrivoruchko, AlexanderXue, FengKroesen, MichielYadav, DhananjayKrol, SilkeYadav, RajKrólicka, AleksandraYadav, Vikash KumarKučera, JanYakimovich, ArturKucharczyk, DariuszYamada, HiroyaKucharz, JakubYamane, KyokoKuchumov, AlexYan, ShanKudryavtsev, Denis S.Yáñez-Rivera, BeatrizKuehn, SandraYang, Byoung-eunKuhbandner, KristinaYang, Chin-Cheng ScottyKuhlman, Thomas E.Yang, ChuanyuKuhn, ElisabettaYang, DingKukreja, ShwetaYang, Jiann-JouKumar, AkhileshYang, Wen-BinKumar, AmitYao, JiayiKumar, AnujYao, Pei-LiKumar, DhirendraYao, QichaoKumar, HirdeshYeh, Chau-TingKumar, ManishYeung, Andy Wai KanKumar, ManuYilmaz, AsliKumar, RajYisraeli, JoelKumar, ShaileshYogeswara, Ida Bagus AgungKumar, VikashYokoi, HayatoKumar, VinodYokose, SatoshiKumari, MeenaYoo, Mi-jeongKumirska, JolantaYork, DanielKummerli, RolfYoshida, ShinichiroKung, Chin-PingYoshida, TadashiKunz, JoachimYoshihara, TatsuyaKuo, Hann-ChorngYoshioka, KentaroKuo, Szu-ChengYoshiyama, MitsuharuKupczyński, RobertYou, Hye JinKurabayashi, AtsushiYoung, JessicaKurowski, MarcinYousefi, Morteza Kurta, AllenYu, FengqunKuryłowicz, AlinaYu, KaiKusari, ArpanYu, NingKutikhin, AntonYu, TengtengKutzsche, JanineYzydorczyk, CatherineKuzmin, KirillZacchia, MiriamKvist, SebastianZadmajid, VahidKwan, Hiu-yeeZahran, EmanKwon, DeukwooZaitsev, AlekseyKwon, Soon-ChanZalevsky, ArturLa Penna, Giovanni Zamljen, TilenLabajova, NadaZamorska, JustynaLabro, AlainZampieri, SandraLacalle, EstíbalizZanotti, GiuseppeLach, SławomirZarębska-Michaluk, DorotaLacomme, ChristopheZarrabi, AliLage, Jean-Luc DaZeltzer, Assaf A.Lai, Ching TatZemanova, MiriamLai, Chyong-HueyZeng, BaoshengLaine, ÉlodieZenteno-Galindo, Arturo EdgarLakin-Thomas, Patricia L.Zernig, GeraldLakota, JanZervou, Fainareti N.Laliotis, George P.Zgair, AtheerLam, Suk YeeZhang, CaixiLamango, Nazarius S.Zhang, HanLamazière, AntoninZhang, KaiLambrughi, MatteoZhang, MingyiLamm-Shalem, NoaZhang, ShixiongLan, Michael S.Zhang, SouquanLanba, AsheeshZhang, TingLancé, MarcusZhang, WeijieLanciers, SophieZhang, YongliangLange, EwaZhang, YuanLangsetmo, Lisa A.Zhao, BingchunLanning, NathanZhao, HaiqingLansdown, RichardZhao, JiananLapping-Carr, GabrielleZhao, LinboLaranjo, MartaZhao, LingxiaLars, GoebelZhou, JianLasoń, WładysławZhou, JunLasonder, EdwinZhou, JunhongLassudrie, MalwennZhou, ShuanhuLászló, SzeredayZhu, ShujiaLatella, GiovanniZienkiewicz, KrzysztofLatino, FrancescaZino, LorenzoLau, KeithZloh, MireLäubli, NinoŽoldák, GabrielLavender, AndrewZou, WeiLavie, Carl J.Zschorlich, Volker RudolfLaVoie, Holly A.Zsolt, KotroczóLavoie, Kathleen HoeyZulkifli, MohammadLaw, DavidŽunar, BojanLaw, Henry Chun HinZuo, HaoLaw, John Lok ManZwart, RebeccaLazarević, Vladimir



